# 

**Published:** 1908-12-26

**Authors:** 


THE HOSPITAL December 26, 1908.

*Name* ..............................

*Address* ..............................

..............................

This Coupon must accompany manuscript or contributions intended for The Hospital.

